# Resistance Mutation Patterns among HIV-1-Infected Children and Features of the Program for Prevention of Mother-to-Child Transmission in Vietnam’s Central Highlands and Southern Regions, 2017–2021

**DOI:** 10.3390/v16050696

**Published:** 2024-04-28

**Authors:** Huynh Hoang Khanh Thu, Alexandr N. Schemelev, Yulia V. Ostankova, Diana E. Reingardt, Vladimir S. Davydenko, Nguyen Tuong Vi, Le Ngoc Tu, Ton Tran, Truong Thi Xuan Lien, Aleksandr V. Semenov, Areg A. Totolian

**Affiliations:** 1 Pasteur Institute in Ho Chi Minh City, Ho Chi Minh City 70000, Vietnam; hhkthupasteur@gmail.com (H.H.K.T.); vinguyentuong1407@gmail.com (N.T.V.); lengoctu1105@gmail.com (L.N.T.); trantonlx@yahoo.com (T.T.); 2 St. Petersburg Pasteur Institute, St. Petersburg 197101, Russia; shenna1@yandex.ru (Y.V.O.); dianavalutite008@gmail.com (D.E.R.); vladimir_david@mail.ru (V.S.D.); totolian@pasteurorg.ru (A.A.T.); 3 Faculty of Pharmacy, Van Lang University, Ho Chi Minh City 70000, Vietnam; lien.ttx@vlu.edu.vn; 4 FSRIVI, “VIROME”, Rospotrebnadzor, Ekaterinburg 620030, Russia; semenov_av@niivirom.ru

**Keywords:** HIV-1, children, prevention of mother-to-child transmission, early infant diagnosis, mutation

## Abstract

The Vietnam Ministry of Health (MOH) has intensified efforts in its aim to eliminate AIDS by 2030. Expanding the program for prevention of mother-to-child transmission (PMTCT) is a significant step towards achieving this goal. However, there are still HIV-exposed children who do not have access to PMTCT services, and some who have participated in the program but still contracted HIV. This study focused on assessing the prevalence and profile of HIV mutations among children under 18 months of age who had recently tested positive for HIV, while gaining insights into the implementation of early infant diagnostic (EID) tests. Between 2017 and 2021, 3.43% of 5854 collected dry blood spot (DBS) specimens from Vietnam’s Central and Southern regions showed positive EID results. This study identified a high prevalence of resistance mutations in children, totaling 62.9% (95% CI: 53.5–72.3). The highest prevalence of mutations was observed for NNRTIs, with 57.1% (95% CI: 47.5–66.8). Common mutations included Y181C and K103N (NNRTI resistance), M184I/V (NRTI resistance), and no major mutations for PI. The percentage of children with any resistance mutation was significantly higher among those who received PMTCT interventions (69.2%; 95% CI: 50.5–92.6%) compared with those without PMTCT (45.0%; 95% CI: 26.7–71.1%) with χ2 = 6.06, *p* = 0.0138, and OR = 2.75 (95% CI: 1.13–6.74). Mutation profiles revealed that polymorphic mutations could be present regardless of whether PMTCT interventions were implemented or not. However, non-polymorphic drug resistance mutations were predominantly observed in children who received PMTCT measures. Regarding PMTCT program characteristics, this study highlights the issue of late access to HIV testing for both mothers and their infected children. Statistical differences were observed between PMTCT and non-PMTCT children. The proportion of late detection of HIV infection and breastfeeding rates were significantly higher among non-PMTCT children (*p* < 0.05). Comparative analysis between children with low viral load (≤200 copies/mL) and high viral load (>200 copies/mL) showed significant differences between the mothers’ current ART regimens (*p* = 0.029) and the ARV prophylaxis regimen for children (*p* = 0.016). These findings emphasize the need for comprehensive surveillance to assess the effectiveness of the PMTCT program, including potential transmission of HIV drug-resistance mutations from mothers to children in Vietnam.

## 1. Introduction

The HIV/AIDS epidemic still remains one of the most significant global public health challenges today despite its onset over four decades ago. According to the recent global estimates by the World Health Organization (WHO) and the United Nations Programme on HIV/AIDS (UNAIDS), approximately 39.0 million [33.1–45.7 million] individuals worldwide were living with HIV at the end of 2022. This includes 1.5 million [1.2–2.1 million] children under the age of 15, with 130,000 (90,000–210,000) new HIV infections among children under 5 years old [1,2]. To effectively address the UNAIDS global strategies to end AIDS, it is crucial to prioritize the elimination of HIV transmission from mothers to their newborns and to ensure the health of the mothers themselves. This can be achieved by increasing access to services related to the prevention of mother-to-child transmission (PMTCT), such as early infant diagnosis (EID) for infants born to HIV-infected mothers immediately after birth. Furthermore, it is important to initiate lifelong antiretroviral therapy (ART) for pregnant women living with HIV and provide antiretroviral (ARV) prophylaxis for children at risk.

According to the United Nations Children’s Fund (UNICEF) fact sheet data, there have been notable advancements in recent years to ensure that women who are not already on lifelong antiretroviral treatment before their current pregnancy are promptly and appropriately started on treatment to reduce the risk of HIV transmission from mothers to their babies during pregnancy, childbirth, and breastfeeding. However, 70% of new HIV infections among children in 2022 were attributed to the fact that the mother did not receive ARV or discontinued it during pregnancy or breastfeeding. This is an alarming trend that highlights the critical importance of ensuring that pregnant women living with HIV have access to and adhere to ART throughout these crucial periods [2].

Another important aspect to consider is the potential consequence of high ART coverage in mothers and the administration of prophylaxis in newborns, which can lead to the development of drug-resistance mutations, particularly in regimens containing non-nucleoside reverse transcriptase inhibitor (NNRTI), a drug class with a low genetic barrier [3,4]. These resistance mutations can prevent the effectiveness of both PMTCT prophylaxis and future treatment options for children [5,6,7]. It is necessary to remain vigilant and proactive in monitoring and addressing these potential consequences to ensure optimal outcomes of both prevention and treatment efforts.

According to UNAIDS, the estimated number of individuals living with HIV in Vietnam was around 250,000 (230,000–270,000) at the end of 2022. Among these individuals, there were approximately 3800 children aged 0–14 years [8]. Since the introduction of the PMTCT program for HIV in 2005, there has been steady expansion and enhancement in both maternal and neonatal health services to target the elimination of HIV infection by 2030. Indeed, PMTCT program coverage in Vietnam has experienced a positive and consistent upward trend. Starting from a coverage of 44% in 2010, it increased to 73% in 2017, and further improved to reach an impressive 90% in 2020 [8,9,10].

Unfortunately, the COVID-19 pandemic had a significant impact on PMTCT program coverage in Vietnam. Due to the restrictions and social distancing measures implemented during that time, the coverage dropped from 90% in 2020 to 75% in 2021. At the end of 2022, it was a little higher at 77%. Similarly, access to EID tests, an essential component of the PMTCT program, also experienced a decline. After expanding from 31% in 2017 to 54.6% in 2020, access to EID tests decreased to 27% in 2021 and 2022. This decrease in EID testing can have negative impacts on PMTCT services affecting infants. In terms of the HIV vertical transmission rate, it decreased from 18.6% in 2010 to 9.7% in 2020. However, it was relatively high in 2022 at 12.77%. This indicates the need for continued efforts to improve access to and utilization of PMTCT services to further reduce transmission rates [8,9,10].

During the study period (2017–2021), Vietnam’s national guidelines for HIV/AIDS management, diagnosis, and treatment [11,12] outlined specific recommendations for EID at various time points, such as 0–2 days, 4–6 weeks, and 9 months. These guidelines emphasized the importance of conducting EID tests within the first two days for newborns to ensure early detection of HIV and prompt intervention. However, if it was not possible to perform the test within this timeframe, the guidelines advised the first EID test should be done at four to six weeks of age or as soon as possible if the child exhibited symptoms of HIV infection.

For ARV prophylaxis and treatment, Vietnam adopted Option B+ per WHO recommendations, involving lifelong ARV treatment for all HIV-infected pregnant and breastfeeding women, regardless of their CD4 cell count or clinical WHO stages. The recommended regimen for HIV-infected pregnant women consists of a triple combination of tenofovir disoproxil fumarate (TDF), lamivudine (3TC), and efavirenz (EFV) to prevent mother-to-child transmission of the virus [13,14]. By the end of 2017, women who were diagnosed as HIV-positive later than the 24th week of pregnancy were advised to take a regimen of TDF + 3TC + RAL (raltegravir).

However, since 2020, a fixed-dose combination of TDF+3TC+DTG (dolutegravir) has been recommended as the preferred regimen for all pregnant women living with HIV. It is worth noting that in practice, EFV continued to be used due to the shortage of regimens containing DTG or RAL in Vietnam. In terms of infant prophylaxis, the guidelines recommend a single dose of nevirapine (sdNVP) given within 6 to 12 weeks of birth for HIV-exposed infants. Since 2018, sdNVP for 6 weeks postpartum has been applied for cases with a low risk of vertical transmission. For infants at a high risk of transmission or those being breastfed, a combination of AZT (zidovudine) plus NVP is used for 6 weeks. If the infant is at high risk of transmission and is being breastfed, the prophylaxis is extended to 12 weeks [11,12,15].

These guidelines indeed demonstrate Vietnam’s commitment to PMTCT of HIV and ensuring positive outcomes for both mother and child. However, it is important to note that there is limited information regarding adherence to and effectiveness of these guidelines in practice. To address this gap in the knowledge, this study aimed to assess the prevalence and profile of HIV mutations among children who tested positive through EID and to gain insights into the implementation of the PMTCT program in Vietnam. Data obtained from this study will provide important insights into the current situation of HIV diagnosis and treatment among infants in Vietnam.

## 2. Materials and Methods

The study protocol received approval from the ethics committees at both the Saint Petersburg Pasteur Institute and the Pasteur Institute in Ho Chi Minh City. This retrospective study utilized stored remnant dried blood spot (DBS) samples collected from the Central Highland and Southern regions of Vietnam. These samples were originally identified as HIV+ through early infant diagnosis (EID) testing conducted on children under 18 months of age between May 2017 and May 2021. Demographic information was obtained from Appendix 1C “The laboratory requisition form for EID” as outlined in the national guidelines for HIV testing [16].

All DBS samples that tested positive for HIV, including confirmation using the COBAS^®^ AmpliPrep/COBAS^®^ TaqMan^®^ HIV-1 Qualitative Test v2.0 (sensitivity 16.5 cps/mL, specificity 99.8%), were stored at −20 °C and subsequently transported to the HIV Laboratory at the Saint Petersburg Pasteur Institute for molecular testing. Total nucleic acid was extracted using the AmpliSens^®^ RIBO-prep kit (Moscow, Russia) and subjected to real-time PCR using the AmpliSens^®^ HCV/HBV/HIV-FL assay (HIV sensitivity 200 cps/mL, HIV specificity 100%).

Samples with real-time PCR cycle threshold (Ct) values less than 31 were genotyped in the protease (PR) region and part of the reverse transcriptase (RT) region (nt 2085–3369 according to GenBank database (K03455.1) isolate HXB2). AmpliSens^®^ HIV-Resist-Seq [17] was used for preparation of samples for sequencing. The kit included reagents for all steps including nucleic acid extraction, amplification, and sequencing reaction. Sequencing reaction products were analyzed using an ABI Prism 3500 genetic analyzer (Applied Biosystems, Waltham, MA, USA). Sequenced fragments from the same specimens were then assembled into consensus using Seqman Pro software (DNASTAR Lasergene Coresuite, V17, DNASTAR, Inc., Madison, WI, USA). Analyzed sequences were interpreted for resistance mutations using the Stanford HIVdb database (V9.1, updated on 2 June 2022) [18]. Mutation profiles were analyzed through creating linear diagrams using Linear Diagram Generator online software [19,20].

Data entry was performed using EpiData V3.1, and analysis was conducted using Stata software V16.0 (StataCorp LP, College Station, TX, USA). To assess the statistical significance of numerical data obtained from paired comparisons based on sample characteristics, either the Poisson exact or Fisher’s exact Chi-squared test was utilized. A probability value of *p* < 0.05 was considered as the threshold for statistical significance.

## 3. Results

Between May 2017 and May 2021, a total of 5854 dry blood spot (DBS) specimens were collected from children under 18 months of age in Vietnam’s Central and Southern regions for EID testing of HIV. Among these specimens, 201 tested positive for HIV, indicating a prevalence of 3.43% (95% CI: 2.97–3.94%) for HIV+ children under 18 months of age during that time period. Of the positive results, 90 samples were classified as group 1, indicating a low HIV-1 viral load. Conversely, 111 samples fell into group 2, signifying an HIV-1 viral load exceeding 200 copies/mL, and underwent sequencing testing to assess HIV drug resistance (DR).

### 3.1. Mutation Profiles among HIV-1 Infected Children under 18 Months of Age

Among the group 2 samples, complete fragments of the protease and reverse transcriptase regions were obtained from 105 samples and were then used to analyze the mutation profiles related to DR. The nucleotide sequences of these HIV-1-infected samples have been deposited in GenBank under the accession numbers OR791296-OR791400. To identify resistance mutations, the Stanford HIVdb algorithm (version 9.1) was used, taking into account both major and minor/accessory mutations. The prevalence of these mutations was calculated accordingly.

A total of 62.9% (95% CI: 53.5–72.3) were identified as having any resistance mutations. The highest prevalence of mutations was observed for NNRTIs, with a proportion of 57.1% (95% CI: 47.5–66.8). Mutation prevalence related to nucleoside reverse transcriptase inhibitors (NRTIs) was 10.5% (95% CI: 4.5–16.4), which was relatively low compared with NNRTIs. The facts that no major protease inhibitor (PI) resistance mutations were detected in the samples and the prevalence of accessory mutations related to PIs was 7.6% (95% CI: 2.5–12.8) suggest a relatively lower occurrence of PI resistance mutations in HIV-infected children under 18 months of age.

Out of these 105 samples with complete fragments, 65 cases representing 61.9% (95% CI: 47.8–78.9) reported having access to PMTCT services. Among them, 45 cases received both maternal and neonatal ARV, indicating a comprehensive approach to preventing transmission. Additionally, 13 children received neonatal prophylaxis only, while 7 individuals received maternal therapy only. The presence of resistance mutations among HIV-1-infected children was analyzed and categorized based on PMTCT interventions, as shown in Table 1. The percentage of children with resistance mutations was significantly higher among those who received PMTCT intervention (69.2%; 95% CI: 50.5–92.6%) compared with those without PMTCT (45.0%; 95% CI: 26.7–71.1%). This difference was significant with an χ2 value = 6.06, *p* = 0.0138, and OR = 2.75 (95% CI: 1.13–6.74).

**Figure 1:**
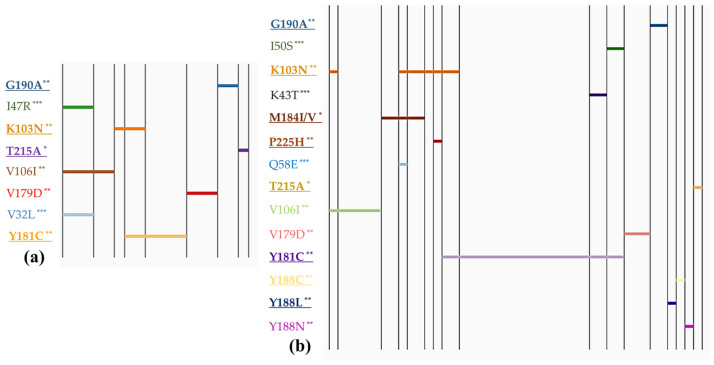
Linear diagrams of multiple mutation profiles. Mutations are distributed along the horizontal axis. The numbers of cases are represented by the length of the horizontal axis. Bold underlines indicate major mutations, *: mutations to NRTIs, **: mutations to NNRTIs, ***: mutations to PIs. (**a**) Children without PMTCT intervention (n = 18) and (**b**) children receiving PMTCT intervention (n = 43).

To facilitate the interpretation of mutation patterns within the study population, linear diagrams were created. Figure 1 depicts the resistance mutation profiles in children, comparing those with and without PMTCT prevention.

**Figure 2:**
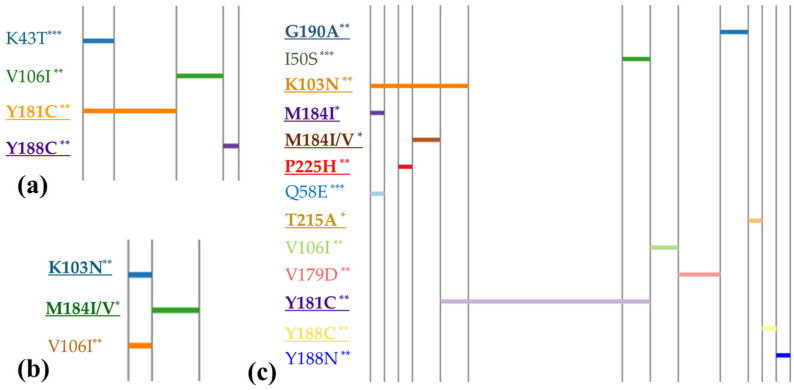
Linear diagrams of multiple mutation profiles along children with PMTCT intervention. Mutations are distributed along the horizontal axis. The numbers of cases are represented by the length of the horizontal axis. Bold underlines indicate major mutations, *: mutations to NRTIs, **: mutations to NNRTIs, ***: mutations to PIs. (**a**) Neonatal prevention only (n = 10), (**b**) maternal prevention only (n = 3), and (**c**) both neonatal and maternal intervention (n = 30).

Figure 1 and Figure 2 categorize these profiles based on different types of intervention. The mutation profiles revealed that polymorphic mutations could be present regardless of whether PMTCT interventions were implemented or not. However, non-polymorphic DR mutations were predominantly observed in children who received PMTCT measures. Statistical analysis showed a significantly higher prevalence of DR mutation accumulation among individuals who received PMTCT interventions (OR = 3.95, 95% CI: 1.56–10.3), compared with those without interventions (χ2 = 10.49, *p* = 0.0012). However, we did not observe any notable difference in the prevalence of polymorphic mutations between the two groups. The major mutation M184I/V (causing resistant to NRTIs) was found in children whose mothers received ART, whereas it was not detected in children who received neonatal interventions only.

Furthermore, our analysis found one HIV-infected child (0.8%) had mutations associated with resistance to triple ARV classes. Additionally, two children (1.9%) were found to carry viruses with mutations conferring resistance to both NRTIs and NNRTIs. Seven cases (6.7%) exhibited mutations resistant to both NNRTIs and PIs. Notably, six children (5.7%) displayed two or more NNRTI mutations. It was observed that most cases with more than two DR mutations belonged to the group of children who received both maternal and neonatal prevention. However, it was also found in two cases where no intervention was reported but which showed accumulation of two NNRTI DR mutations.

### 3.2. Characteristics Related to PMTCT of HIV among Children Who Had Recently Been Diagnosed as HIV-1 Positive through EID Testing

Out of 201 HIV+ samples, 116 infants (57.7%) were female, and only 44 infants (21.9%) resided in Ho Chi Minh City (HCMC). The majority of the children, comprising 78.1% (95% CI: 66.4–91.3%) of the total, came from the provinces, with 127 from the Southern region and 30 from the Central and Highland regions of Vietnam. This study’s findings revealed that a low percentage of children among recently diagnosed HIV-infected individuals had accessed the EID testing recommended by the MOH’s guidelines [11,12]. On average, the duration from the child’s birth to the date of collecting DBS specimens for EID testing was 14.7 weeks, with a median duration of 8.3 weeks. Among these children, up to 56.2% (95% CI: 46.3–67.6%) were between 6 weeks and less than 9 months old at the time of specimen collection. Regarding notification, 15.9% (95% CI: 10.9–22.5%) received a diagnosis within one week; 5.0% (95% CI: 2.4–9.1%) within four weeks; and 14.4% (95% CI: 9.7–20.7%) within 4–6 weeks after birth.

Notably, there was a difference in this characteristic between children who received PMTCT interventions and those who did not. Among children who received PMTCT, 47.7% (95% CI: 32.4–67.7%) underwent EID testing within the first six weeks, with mean and median times of 9.8 and 6.1 weeks, respectively. However, for children without PMTCT, only 20% underwent testing within this timeframe, with mean and median times of 20.4 and 16.8 weeks, respectively. This difference was statistically significant (χ2 = 8.13, *p* = 0.002, OR = 3.65, 95% CI: 1.36–6.73).

Similarly, the study highlighted a high percentage (71.1%, 95% CI: 60.0–83.8%) of mothers who were either diagnosed late with HIV or had unknown infection status. Out of the 201 cases, 73 (36.3%) were detected during labor, while 70 cases (34.8%) were identified at a later stage when the children were hospitalized, or their HIV status remained unknown due to the children being abandoned. Again, there was a significant difference in this characteristic between the PMTCT and non-PMTCT groups. In the PMTCT group, 93.8% of children had mothers who were diagnosed with HIV infection at certain points (prior to pregnancy, during pregnancy, or at labor), compared with only 17.5% in the non–PMTCT group (χ2 = 63.25, *p* < 0.001, OR = 71.9).

The analysis of PMTCT characteristics among HIV-1-infected children under 18 months of age included various factors related to PMTCT interventions, as shown in Table 2. A quite high proportion of mothers (44.3%, 95% CI: 35.6–54.5%) had not yet received any ARV regimen. A large proportion of the mothers (82.1%, 95% CI: 64.9–99.8%) were being treated with ARV using regimens featuring two NRTIs and one NNRTI. In terms of child ARV prophylaxis, 69.9% (95% CI: 55.3–87.1%) received an sdNVP. The mean duration of ARV prophylaxis for children until the date of collecting the sample was 64.8 days, with a range of 1 to 307 days.

Regarding feeding practices, only 21.9% (95% CI: 15.9–29.3%) of the children were raised through breastfeeding, while the remaining children relied on formula milk or a combination of breastfeeding and formula feeding. Comparative analysis was also conducted on feeding practices among the two groups (PMTCT, non–PMTCT). It was notable that 50.0% of children without PMTCT were breastfed, compared with only 6.2% of children with PMTCT (χ2 = 17.66, *p* < 0.001, OR = 10.25).

Our study also assessed EID test turnaround time (TAT). The mean time between the collection of DBS specimens and the delivery of results to caregivers was 10.4 days (95% CI: 9.1–11.7), whereas the mean time between the DBS being received at the laboratory and the EID report being sent to caregivers was 4.4 days (95% CI: 3.9–5.1).

In the comparative analysis examining demographics and PMTCT features between the two groups, significant differences were observed in two specific regimens: the mother’s current ART regimen (*p* = 0.029) and the ARV prophylaxis regimen for children (*p* = 0.016). Further analysis was conducted to investigate the specific ARV regimens and prophylaxis that contributed to the observed significant differences. Interestingly, a higher likelihood of receiving the AZT/3TC/NVP regimen in group 1 was found compared with group 2, with χ2 = 4.51, *p* = 0.025, and OR = 3.22 (95% CI: 0.95–12.47%). However, it is important to note that the sample size for some regimens might be insufficient to conduct such analysis further.

## 4. Discussion

The effectiveness of PMTCT of HIV is measured through EID procedures using nucleic acid testing (NAT). The majority of studies targeted to assess this effectiveness have been based in African countries (77%), with fewer from low- and middle-income countries (LMICs) in Asia (16%) and the Americas (the Caribbean, Central America, and South America; 7%) [21]. In Vietnam, there are limited national data regarding the effectiveness of the PMTCT program for HIV among children under 18 months of age. The most recent literature on this topic was published in 2019 about the PMTCT results from 2017 [22]. Additionally, there is also limited information on DR among this population who were vertically infected with HIV from their mothers. This is the first study investigating both PMTCT program features and the DR situation among early-HIV-diagnosed infants. The HIV National Reference Laboratory at the Pasteur Institute in HCMC is responsible for conducting all EID tests from the Central Highland to the Southern regions of Vietnam. Although this study did not involve national surveillance efforts, the DBS samples collected represent two out of three national regions.

The HIVDR Report 2021 by the WHO highlights that only 10 countries, all from Africa and with a high prevalence of HIV infection, provided data from surveys conducted between 2012 and 2020 [23]. Our study revealed a high percentage of children infected with HIV were found to have harbored resistance mutations, with an overall prevalence of 62.9%. The highest prevalence was observed for NNRTIs (57.1%), followed by 10.5% for NRTIs and 7.6% for accessory mutations associated with PIs. Notably, no major mutations resistant to PIs were detected in the analyzed samples. These finding are in contrast with the previous literature [5,23,24,25], as our study aimed to comprehensively understand mutation profiles by assessing both major and minor/accessory mutations, whereas others reported mutations conferring resistance at low, intermediate, or high levels. However, when comparing our results with similar interpretations from other studies, we found consistent findings regarding the percentage of mutations observed. For instance, a survey conducted in Haiti (2013–2014) examined 304 newly HIV-infected children and revealed that 71.4% of the children had at least one DR mutation. The highest rate of mutations was observed for NNRTIs at 69.1%, followed by NRTIs at 40.5% [26]. A comparative analysis reveals that the prevalence reported in the Haitian study was significantly higher than that reported in our study (*p* < 0.05 for all Chi-square tests on comparing the proportion of any mutation, NNRTI, and NRTI).

In a separate national survey conducted in Togo (2012–2013), percentages of 60% for any mutation and 49.3% for NNRTI were reported. Among children with reported exposure to maternal and/or infant ARVs, mutations were detected in 75.6% of children [27]. In our study, the proportion of any mutation among children with PMTCT was found to be 69.2%, which was lower than the findings observed in Togo’s surveillance.

Our study revealed a significant difference in the percentage of children with resistance mutations between those who received PMTCT intervention (69.2%) and those who did not (45.0%). This indicates that children who underwent PMTCT intervention had a higher likelihood of developing resistance mutations. The difference observed between the two groups (PMTCT, non–PMTCT) was also significant, as evidenced by a χ2 value of 6.06 and a *p*-value of 0.0138. Additionally, our results show that children who received PMTCT intervention were 2.75-fold more likely to develop resistance mutations compared with those without PMTCT. These suggest that the association between PMTCT intervention and the occurrence of resistance mutations is unlikely to be due to chance alone.

The highest prevalence of mutations observed in our study can be attributed to the selective pressure imposed by the use of NNRTI-based regimens in both the mothers and children. NNRTIs have a low genetic barrier, which means they are more prone to causing DR compared with other classes of ARV. In our study, the average time from birth to the collection of DBS specimens among children with PMTCT was 9.8 weeks, with a large number of children using ARV prophylaxis in the first 6 weeks. This suggests that mutations can occur within a relatively short period of time or be transmitted from mother to child.

In addition, the relatively low prevalence of mutations related to NRTIs compared with NNRTIs may be attributed to the limited number of children receiving prophylaxis regimens containing NRTIs. However, we cannot completely rule out the possibility the child acquired the mutations from his/her mother who was on ART. The drug-resistance mutations observed among the children could have occurred either through the development of de novo resistance during the prophylactic period or through transmission from mothers with viral infections featuring DR mutations. Unfortunately, our study, which used remnant DBS specimens from EID testing, did not ascertain the status of HIV DR among the mothers of the children included. To gain insight into this aspect, we conducted an additional study in 2021 among HIV-infected pregnant women who were receiving PMTCT in HCMC. The findings from that study revealed that resistance mutations for any drug were detected in 74.41% of the patients (95% CI: 62.71–85.54%). Specifically, 44.26% of patients exhibited resistance mutations to NNRTIs, while only 8.2% exhibited resistance mutations to NRTIs [28]. These prevalence values were similar to those in children. To better comprehend the transmission of mutations from mother to child, it is essential to examine mutation profiles.

Our study showed a significantly higher prevalence of major mutation among individuals who received PMTCT interventions compared with those without PMTCT, whereas polymorphic and non-polymorphic accessory mutations could be present regardless of PMTCT interventions. Children who underwent PMTCT interventions had a 3.95-fold higher risk of DR mutation accumulation compared with those who did not receive interventions. In terms of major resistance mutations identified in our study, these included several to NNRTIs, including K103N, Y181C, Y188L/C, G190A, and P225H; M184I/V and T215A to NRTI; and none to PIs. Among these, Y181C and K103N were the most common mutations associated with NNRTI resistance, while M184I/V was the most common mutation associated with NRTI resistance. Importantly, these mutations were also observed as the most frequent mutations among pregnant women in our previous study [28], as well as in adults with ART pretreatment and those under ART in Vietnam [29,30,31].

Furthermore, these mutations were found to be highly prevalent among infants newly diagnosed with HIV in 10 other representative African surveys conducted between 2011 and 2018 [6,23]. It is important to note that NRTI resistance mutations were only detected in children who had received maternal ARV treatment, either alone or in combination with neonatal interventions. This suggests a potential transmission of these mutations from the mother to her child.

A case–control study (2011 to 2016) was conducted at 14 sites in seven countries (India, Malawi, South Africa, Tanzania, Uganda, Zambia, Zimbabwe) to assess whether maternal HIV DR was associated with an increased risk of vertical transmission, and to examine the dynamics of DR in HIV–infected infants. The study revealed that, initially, at the time of diagnosis, HIV DR was less prevalent among infants with in utero/peripartum transmission compared to those with breastfeeding vertical transmission. However, over time, DR emerged in both groups [32]. This suggests that the administration of NVP, whether to the mother during delivery or to the infant during breastfeeding, probably played a role in the emergence of DR.

It is noteworthy that the majority of mother–infant pairs that exhibited a discrepancy in their genetic profiles at diagnosis had maternal wild-type HIV, indicating that the infection occurred during breastfeeding. It is plausible that these infants were initially infected with wild-type HIV, and subsequent NVP prophylaxis contributed to the selection of DR mutations, which were then detected in their diagnostic specimens. These findings imply that NVP prophylaxis may have played a significant part in the development of DR in these infants [32]. Thus, the same mutation profiles between pregnant women and children under 18 months of age in our two studies may imply the transmission of HIV from mother to child, especially in those who are breastfeeding.

Furthermore, it is worth mentioning that the total number of major mutations identified in our study was lower compared with the aforementioned studies. For instance, we did not observe mutations such as L100IV, K101EHP, or V106AM, which are associated with resistance to NNRTIs. Additionally, infants surveyed in Africa presented mutations M41L, K65R, and some thymidine analogue mutations (TAMs) (D67NE, K70R, K219QE) that were not observed in our study [6,23]. Regarding non-polymorphic accessory and polymorphic mutations, an interesting finding in our study was the absence of any associated with NRTIs. In contrast, several surveillance studies in Africa have reported the presence of accessory TAMs, such as E40F, E44D/A, and V118I, which are known to contribute to decreased NRTI susceptibility, particularly when combined with multiple other TAMs. Similarly, we also found fewer accessory and polymorphic mutations associated with NNRTIs compared with other studies. In the case of PIs, we discovered a similar mutation pattern to the studies conducted in Africa [23,26]. Furthermore, our analysis revealed that some children had two to three mutations that conferred resistance to different ARV classes. While the prevalence of children with single mutations was higher, the presence of multiple mutations suggests a potentially negative impact on ARV treatment outcomes among these HIV-infected children.

Although our study found a lower number of mutations among HIV-infected children under 18 months of age in Vietnam compared with previous studies, the high percentage of any mutation still poses challenges to the effectiveness of ART in recently detected vertical transmission cases. Especially, NVP has a low genetic barrier to resistance, and even short-term exposure to the drug can lead to the selection of resistant variants [33,34]. This finding sheds light on the importance of considering alternative and more potent ART regimens to reduce vertical transmission and address the challenges posed by DR in the treatment of HIV-infected children, particularly in cases where maternal pretreatment DR to NNRTIs is prevalent.

Regarding insights on the implementation of the PMTCT program in Vietnam, our findings indicate the rate of vertical transmission of HIV among children under 18 months of age, as determined by EID testing between 2017 and 2021, was 3.43%. This rate is lower than the reported data from 2017 in the same study population, which recorded a rate of 4.5%. These results confirm the decreasing trend in the transmission rate noted by Tran Ton et al., who observed a decline in this rate since 2013, particularly since 2015 (*p* = 0.03) [22]. This suggests that efforts to implement the HIV PMTCT program are having a positive impact in Vietnam.

This study provides insights into both strengths and weaknesses in the current intervention program. Among HIV-1-infected children, a significant proportion (up to 56.2%) were diagnosed within 6 weeks to 9 months, with a median duration of 8.3 weeks (58 days). It is noteworthy that the duration was considerably shorter (6.1 weeks or 42.7 days) in children who received PMTCT interventions, compared with those who did not (16.8 weeks or 118 days), which indicates delays in testing and may have resulted in delayed initiation of ART. These findings can be compared with data reported by the WHO, which indicated the mean age at infant testing for HIV was 74 days [14,35]. The high percentage (71.7%) of late detection of HIV infection among mothers was also determined among HIV-infected children. This can increase the risk of vertical transmission, while delaying the implementation of preventive measures. However, it would have been beneficial to compare the data concerning PMTCT interventions among HIV-infected children with those from children exposed to HIV who yet had negative HIV NAT results. Unfortunately, this was a limitation of our study. The findings of Tran Ton et al.’s study, which reported data from both HIV-negative children and those with HIV infection in the same study population as ours, revealed a similar scenario in terms of EID access. Their research showed that 59.18% of HIV-infected children under 18 months of age were diagnosed within 6 weeks to 9 months, consistent with our finding of 56.2%. In contrast, this prevalence among children exposed to HIV who had negative results for HIV NAT was much lower at 30.0%. Importantly, Tran Ton et al. found that late detection of HIV in children after 8 weeks and in mothers during labor was associated with a significantly higher risk of HIV vertical transmission (*p* < 0.001) [22].

These findings emphasize the critical need for improved antenatal screening protocols and increased awareness about the importance of HIV testing for expectant mothers and intervention to improve outcomes for both mothers and children affected by HIV. Additionally, our study revealed a significant difference in early HIV detection between mothers who fully or partially participated in the PMTCT program compared with non–PMTCT individuals (χ2 = 63.25, *p* < 0.001, OR = 71.9). This finding reflects the effectiveness of PMTCT programs in increasing awareness and facilitating access to early testing services. In terms of breastfeeding, there was a statistically lower rate of breastfeeding observed in children who underwent PMTCT compared with those who did not receive PMTCT interventions. This stark difference suggests that mothers who received PMTCT services were more likely to opt for alternative feeding methods, such as formula milk.

The decision to avoid breastfeeding in the PMTCT group is primarily due to the risk of HIV transmission through breast milk, as there is a possibility of the virus being present in breast milk even if the mother is on ART. However, it is worth noting that there were instances of vertical HIV transmission from mother to child even among those who participated in the PMTCT program. This finding underscores the need for further research to gain insights into the reasons behind HIV transmission despite PMTCT participation. By understanding these factors, we can improve the effectiveness of interventions and provide better support to prevent vertical transmission and ensure the health and wellbeing of both mothers and children affected by HIV.

In addition, TAT from sample collection to the release of EID reports is another crucial factor that can impact the effectiveness of PMTCT programs. Our study showed significantly better results compared with a systematic review conducted by the WHO in 2021. Our findings indicated a mean TAT of 10.4 days (95% CI: 9.1–11.7 days), with the time from DBS delivery at the laboratory to the caregiver receiving the results being only 4.4 days (95% CI: 3.9–5.1). For the WHO’s traditional approach for EID testing using HIV NAT, the median time from sample collection to the caregiver receiving the result was 35 days (range 8–125 days, 95% CI 35–37 days). Five out of seven studies reported a median time of more than 30 days for caregivers to receive the results [14,36,37].

These findings highlight the remarkable efforts made by the Vietnam MOH in expanding EID testing for children and ensuring swift connections with care and treatment programs. However, data from point-of-care (POC) testing demonstrate even better results. Same-day POC testing significantly reduces the time taken to deliver results to caregivers (high-certainty evidence). Across all seven studies, the median time from sample collection to results being received by the infants’ caregivers was 0 days (95% CI: 0–0 days) when using POC testing, regardless of the test used, infant age, or type of healthcare facility. POC testing returned same-day results in 97% of cases, compared with 0% for the standard of care [14,36]. Considering these impressive outcomes, it is crucial to expedite the implementation of POC HIV NAT testing in Vietnam to further enhance the effectiveness of PMTCT programs and improve access to ART.

The treatment regimens for mothers and the prophylaxis for children in this study followed the national guidelines [11,12]. Our study revealed that children with an HIV-1 viral load lower than 200 copies/mL at the time of DBS collection were more likely to receive the AZT/3TC/NVP regimen compared with those with a high HIV-1 viral load. However, the number of infants using this regimen was relatively small.

Thus, despite the recommendation in Vietnam’s national guidelines for HIV treatment and care (Decision No 3047/QD-BYT, dated 22/07/2015) [15] to perform early diagnosis tests on infants within 4–6 weeks, and as soon as possible within the first two days after birth (Decision N0 5456/QD-BYT, dated 20/11/2019) [12], this study found that a number of children under 18 months of age who tested positive for HIV through EID had not had their tests administered in accordance with the guidelines. This misalignment could have a negative impact on Vietnam MOH’s strategy to eliminate new pediatric HIV infections by 2030. Undetected HIV infection progresses rapidly in infants, with 30% of undiagnosed infants dying before their first birthday and 80% dying before the age of 5. EID allows prompt ART initiation and improves the survival of infants living with HIV [38]. Similar challenges in accessing early HIV diagnosis have been reported in other resource-limited settings [39,40].

There are several limitations to consider when interpreting the findings of our study. Firstly, during the study period, the coverage of EID testing for HIV in Vietnam was estimated to be around 60% according to UNAIDS [8]. This implies that there is a possibility of underdiagnosis of HIV in many children, which could potentially impact the results and conclusions of our study. Additionally, our report reflects only the remnant DBS samples collected from Central Highlands and Southern Vietnam. As such, it may not provide a comprehensive representation of the entire population of children under 18 months infected with HIV in the country. However, it does provide valuable insights for two-thirds of Vietnam’s regions.

To assess the effectiveness of the HIV PMTCT program, it would be beneficial to conduct a comparative analysis on characteristics related to the program for both HIV-exposed yet uninfected children and HIV-infected children. However, we were unable to obtain data on the former group, thus limiting our ability to make such a comparison. Furthermore, our study lacks information regarding the extent of HIV DR among mothers. This limitation stems from our methodology, which relied on stored remnant DBS samples and data collected during routine national EID activity for children under 18 months of age. Consequently, we were not able to specifically assess or report on HIV DR in the mothers.

## 5. Conclusions

In conclusion, this study’s findings highlight the gaps and challenges in the implementation of EID testing and PMTCT interventions in Vietnam. They emphasize the need for comprehensive strategies to improve access to early HIV diagnosis, while promoting timely testing at birth or at least within six weeks of life. Collaboration between healthcare providers, policymakers, and communities is essential to address these challenges and to reduce the burden of pediatric HIV in Vietnam. Furthermore, it is recommended that further studies be conducted to examine pretreatment DR infection status among pregnant women and DR transmission from mother to child in mother–infant pairs. Such studies will help us gain a better understanding of DR transmission and to develop appropriate strategies to mitigate its impact. By implementing these measures, we can enhance the long-term outcomes for HIV-infected children and provide improved care for their overall health and wellbeing.

## Figures and Tables

**Table 1:** Resistance mutations among HIV-1-infected children, categorized by PMTCT intervention. Bold underlines indicate major mutations.

ARV Class	Mutation	Children without PMTCT (N = 40)		Children with PMTCT (N = 65)				
		n	%	Neonatal Only (n = 13)	Maternal Only (n = 7)	Both Neonatal and Maternal (n = 45)	Total Number	%
Any mutation		18	45.0%	11	3	31	45	69.2%
PI	V32L	3	7.5%					
	I47R	3	7.5%					
	K43T			2			2	3.1%
	I50S					2	2	3.1%
	Q58E					1	1	1.5%
NNRTI	**K103N**	3	7.5%		1	7	8	12.3%
	V106I	5	12.5%	3	1	2	6	9.2%
	V179D	3	7.5%			3	3	4.6%
	**Y181C**	6	15.0%	6		15	21	32.3%
	**Y188L**					1	1	1.5%
	**Y188C**			1			1	1.5%
	Y188N					1	1	1.5%
	**G190A**	2	5.0%			2	2	3.1%
	**P225H**					1	1	1.5%
NRTI	**M184I/V**				2	3	5	7.7%
	**T215A**					1	1	1.5%

**Table 2:** PMTCT characteristics among HIV-1-infected children under 18 months of age.

**Characteristic**	**Total (N)**	**n**	**%**
ART situation of mothers prior to or when giving birth	201		
Not yet		89	44.3
ART or PMTCT		95	47.3
Missing		17	8.5
Mother’s current ART regimen	95		
TDF/3TC/EFV		76	80.0
TDF/3TC/LPVr		14	14.7
AZT/3TC/EFV		2	2.1
TDF/3TC/DTG		3	3.2
ARV prophylaxis for child	201		
No		85	42.3
Yes		113	56.2
Unknown		3	1.5
Child’s prophylaxis regimen	113		
NVP		79	69.9
NVP/AZT		11	9.7
AZT/3TC/NVP		17	15.0
AZT		1	0.9
AZT/3TC		3	2.7
Missing		2	1.8
Duration of ARV prophylaxis for child until date of collecting sample	113		
Mean (days)		64.8	
Median (days)		42	
Variant (day)		0–307	
Babies were raised with	201		
Breastfeeding		44	21.9
Formula milk		146	72.6
Both breastfeeding and formula milk		5	2.5
Unknown		6	3.0

## Data Availability

Data are available on request from the authors.

## References

[1] World Health Organization HIV Data and Statistics. https://www.who.int/teams/global-hiv-hepatitis-and-stis-programmes/hiv/strategic-information/hiv-data-and-statistics.

[2] UNICEF Elimination of Mother-to-Child Transmission. UNICEF, July 2023. https://data.unicef.org/topic/hivaids/emtct/.

[3] Luber A.D. (2005). Genetic barriers to resistance and impact on clinical response. Afr. J. Reprod. Gynaecol. Endosc..

[4] Mackie N., Geretti A.M. (2006). Resistance to non-nucleoside reverse transcriptase inhibitors. Antiretroviral Resistance in Clinical Practice.

[5] Hitti J., Halvas E.K., Zheng L., Panousis C.G., Kabanda J., Taulo F., Kumarasamy N., Pape J.W., Lalloo U., Sprenger H. (2014). Frequency of Antiretroviral Resistance Mutations among Infants Exposed to Single-Dose Nevirapine and Short Course Maternal Antiretroviral Regimens: ACTG A5207. J. AIDS Clin. Res..

[6] Jordan M.R., Penazzato M., Cournil A., Vubil A., Jani I., Hunt G., Carmona S., Maphalala G., Mthethwa N., Watera C. (2017). Human Immunodeficiency Virus (HIV) Drug Resistance in African Infants and Young Children Newly Diagnosed with HIV: A Multicountry Analysis. Clin. Infect. Dis..

[7] Rojas Sánchez P., Holguín A. (2014). Drug resistance in the HIV-1-infected paediatric population worldwide: A systematic review. J. Antimicrob. Chemother..

[8] UNAIDS (2022). UNAIDS DATA 2022. https://www.unaids.org/en/resources/documents/2023/2022_unaids_data.

[9] UNAIDS (2018). UNAIDS Data 2018. https://www.unaids.org/en/resources/documents/2018/unaids-data-2018.

[10] UNAIDS (2021). UNAIDS Data 2021. https://www.unaids.org/en/resources/documents/2021/unaids-data-2021.

[11] Vietnam Ministry of Health (2017). National Guidelines for HIV/AIDS Management, Diagnosis and Treatment.

[12] Vietnam Ministry of Health (2019). National Guidelines for HIV/AIDS Management, Diagnosis and Treatment.

[13] WHO-Guideline (2016). Consolidated Guidelines on the Use of Antiretroviral Drugs for Treating and Preventing HIV Infection: Recommendations for a Public Health Approach. https://www.who.int/publications/i/item/9789241549684.

[14] WHO-Guideline, 7/2021, “Consolidated Guidelines on HIV Prevention, TESTING, Treatment, Service Delivery and Monitoring: Recommendations for a Public Health Approach,” (Electronic Version). ISBN 978-92-4-003159-3. https://www.who.int/publications/i/item/9789240031593.

[15] Vietnam Ministry of Health (2015). National Guidelines for HIV/AIDS Management, Diagnosis and Treatment.

[16] Vietnam Ministry of Health (2018). National Guideline for HIV Testing.

[17] Manual of HIV-Resist-Seq (RU).

[18] Stanford University HIV Drug Resistance Database. https://hivdb.stanford.edu/hivdb/by-sequences/.

[19] Gottfried B. (2015). A Comparative Study of Linear and Region Based Diagrams. J. Spat. Inf. Sci..

[20] University of Kent. https://www.cs.kent.ac.uk/people/staff/pjr/linear/.

[21] Carlucci J.G., Liu Y., Friedman H., Pelayo B.E., Robelin K., Sheldon E.K., Clouse K., Vermund S.H. (2018). Attrition of HIV-exposed infants from early infant diagnosis services in low- and middle-income countries: A systematic review and meta-analysis. J. Int. AIDS Soc..

[22] Ton T., Vi N.T., Anh L.Q., Hoa H.T.H., Quang P.P., Thinh V.X. (2019). Results of HIV-1 Early Infant Diagnosis among children under 18 months from hue Southward in 2017 at national reference HIV laboraotry of Pasteur Institute in Ho Chi Minh City. Vietnam. J. Prev. Med..

[23] WHO Technical Report HIV Drug Resistance Report 2021. 2021. (Electronic Version) ISBN 978-92-4-003860-8. https://www.who.int/publications/i/item/9789240038608.

[24] Hunt G.M., Ledwaba J., Salimo A., Kalimashe M., Dinh T.H., Jackson D., Sherman G., Puren A., Ngandu N.K., Lombard C. (2019). Prevalence of HIV-1 drug resistance amongst newly diagnosed HIV-infected infants age 4–8 weeks, enrolled in three nationally representative PMTCT effectiveness surveys, South Africa: 2010, 2011–2012 and 2012–13. BMC Infect. Dis..

[25] Dambaya B., Fokam J., Ngoufack E.S., Takou D., Santoro M.M., Této G., Beloumou G.A., Mouafo L.C.M., Kamgaing N., Sosso S.M. (2020). HIV-1 Drug Resistance and Genetic Diversity among Vertically Infected Cameroonian Children and Adolescents. Explor. Res. Hypothesis Med..

[26] Louis F.J., Segaren N., Desinor O., Beard R.S., Jean-Louis R., Chang J., Boisson S., Hulland E.N., Wagar N., DeVos J. (2019). High Levels of HIV-1 Drug Resistance in Children Who Acquired HIV Infection Through Mother to Child Transmission in the Era of Option B+, Haiti, 2013 to 2014. Pediatr. Infect. Dis. J..

[27] Salou M., Butel C., Konou A.A., Ekouevi D.K., Vidal N., Dossim S., Lawson-Evi K., Nyasenu Y.T., Singo-Tokofaï A., d’Almeida S. (2016). High Rates of Drug Resistance among Newly Diagnosed HIV-Infected Children in the National Prevention of Mother-to-Child Transmission Program in Togo. Pediatr. Infect. Dis. J..

[28] Ostankova Y.V., Shchemelev A.N., Thu H.H.K., Davydenko V.S., Reingardt D.E., Serikova E.N., Zueva E.B., Totolian A.A. (2023). HIV Drug Resistance Mutations and Subtype Profiles among Pregnant Women of Ho Chi Minh City, South Vietnam. Viruses.

[29] Ngo-Giang-Huong N., Huynh T.H.K., Dagnra A.Y., Toni T.D., Maiga A.I., Kania D., Eymard-Duvernay S., Peeters M., Soulie C., Peytavin G. (2019). Prevalence of pretreatment HIV drug resistance in West African and Southeast Asian countries. J. Antimicrob. Chemother..

[30] Dat V.Q., Duong B.D., Nhan D.T., Hai N.H., Anh N.T.L., Thu H.H.K., Ton T., Anh L.Q., Nghia N.T., Thuong N.V. (2018). Viral load suppression and acquired HIV drug resistance in adults receiving antiretroviral therapy in Viet Nam: Results from a nationally representative survey. West. Pac. Surveill. Response J..

[31] Dat V.Q., Anh N.T.L., Van Nghia K., Linh N.T., Thu H.H.K., Tam T.T.M., Ton T., Anh L.Q., Phuc N.D., Huong P.T.T. (2022). The prevalence of pre-treatment and acquired HIV drug resistance in Vietnam: A nationally representative survey, 2017–2018. J. Int. AIDS Soc..

[32] Boyce C.L., Sils T., Ko D., Wong-On-Wing A., Beck I.A., Styrchak S.M., DeMarrais P., Tierney C., Stranix-Chibanda L., Flynn P.M. (2022). Maternal Human Immunodeficiency Virus (HIV) Drug Resistance Is Associated with Vertical Transmission and Is Prevalent in Infected Infants. Clin. Infect. Dis. Off. Publ. Infect. Dis. Soc. Am..

[33] Persaud D., Bedri A., Ziemniak C., Moorthy A., Gudetta B., Abashawl A., Mengistu Y., Omer S.B., Isehak A., Kumbi S. (2011). Slower clearance of nevirapine resistant virus in infants failing extended nevirapine prophylaxis for prevention of mother-to-child HIV transmission. AIDS Res. Hum. Retroviruses.

[34] Fogel J.M., Mwatha A., Richardson P., Brown E.R., Chipato T., Alexandre M., Moodley D., Elbireer A.P., Mirochnick M., George K. (2013). Impact of maternal and infant antiretroviral drug regimens on drug resistance in HIV-infected breastfeeding infants. Pediatr. Infect. Dis. J..

[35] Penazzato M., Revill P., Prendergast A.J., Collins I.J., Walker S., Elyanu P.J., Sculpher M., Gibb D.M. (2014). Early infant diagnosis of HIV infection in low-income and middle-income countries: Does one size fit all?. Lancet Infect. Dis..

[36] Vojnov L., Havlir D., Myer L., Abrams E., Jani I. (2022). Same-day test and treat for infants with HIV infection: Finally within reach. J. Int. AIDS Soc..

[37] Luo R., Fong Y., Boeras D., Jani I., Vojnov L. (2022). The clinical impact of point-of-care infant diagnosis for HIV: A systematic review and meta-analysis. Lancet.

[38] Okusanya B., Kimaru L.J., Mantina N., Gerald L.B., Pettygrove S., Taren D., Ehiri J. (2022). Interventions to increase early infant diagnosis of HIV infection: A systematic review and meta-analysis. PLoS ONE.

[39] Kamble S., Gawde N., Goel N., Thorwat M., Nikhare K., Bembalkar S., Kamble S., Brahme R., Pawar S., Sahoo R. (2023). Access, timeliness and retention for HIV testing under early infant diagnosis (EID) program, India. Sci. Rep..

[40] Nikhare K., Gawde N., Kamble S., Goel N., Kamble S., Pawar S., More P., Kapoor N., Verma V., Kushwaha B.S. (2024). Caregivers’ experiences of accessing HIV Early Infant Diagnosis (EID) services and its barriers and facilitators, India. BMC Health Serv. Res..

